# Development of a new culture medium for bioflocculant production using chicken viscera

**DOI:** 10.1016/j.mex.2019.06.002

**Published:** 2019-06-13

**Authors:** Jibrin Ndejiko Mohammed, Wan Rosmiza Zana Wan Dagang

**Affiliations:** a Department of Microbiology, Ibrahim Badamasi Babangida University, PMB 11, Lapai, Nigeria; b Department of Biosciences, Faculty of Science, Universiti Teknologi Malaysia, Johor Bahru, Malaysia

**Keywords:** Synthesis of low-cost media for bioflocculant production, Hydrolysate, Low-cost, Substrate, Bioflocculation, Efficiency

## Abstract

The economy of mass bioflocculant production and its industrial application is couple with the cost of production. The growth medium is the most significant factor that accounts for the production cost. In order to find a substitute for the expensive commercial media mostly the carbon and nitrogen sources used for bioflocculant production, we use chicken viscera as a sole source of nutrient for bioflocculant production. The culture conditions for *Aspergillus flavus* S44-1 growth and bioflocculant yield were optimized through one factor at a time (OFAT). The use of chicken viscera as a sole source to develop a culture medium seems to be more appropriate, simple, reduce cost of bioflocculant production and in addition offers a sustainable means of managing environmental pollution by the poultry waste. In this article, we focus on detailed description of the steps involve in developing an optimized culture medium using chicken viscera as a sole source for bioflocculant production.

• A new media for bioflocculant production was developed from chicken viscera.
• The culture conditions for bioflocculant production were determined and optimized.
• The bioflocculant yield and efficiency were parallel to mycelial weight at log phase.

## Graphical abstract

**Figure:**
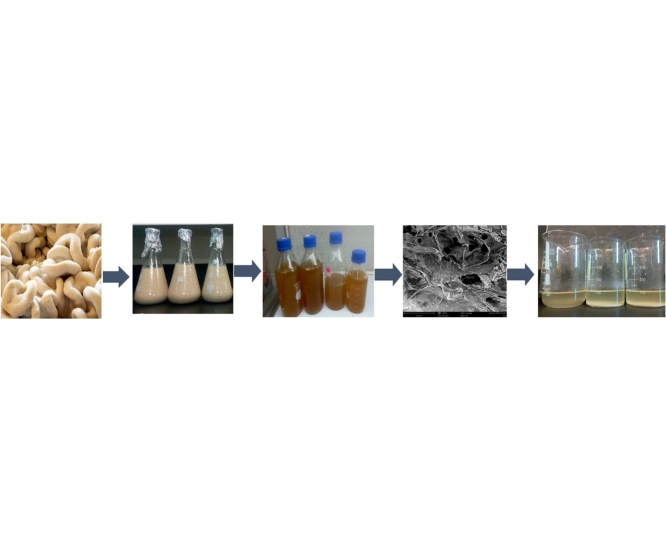

**Specifications Table**

**Table:** 

Subject Area:	Agricultural and Biological Sciences
More specific subject area:	Microbiology
Method name:	Synthesis of low-cost media for bioflocculant production
Name and reference of original method:	N/A
Resource availability:	N/A

## Method details

Briefly, the development of the new culture media from chicken viscera for bioflocculant production as substitute of commercial media involved the following steps:

(1) Collection and processing of the chicken viscera.
(2) Hydrolysis of the processed chicken viscera.
(3) Optimization of the culture conditions
(4) Purification of the bioflocculant and estimation of the bioflocculation activity of the bioflocculant produced from the new culture media

### Collection and processing of the chicken viscera

Chicken viscera (chicken intestine with intestine content) was collected from wet market (Ayam Kempas Sdn Bhd, Johor). The chicken viscera was immediately transported to the laboratory in iced condition. The chicken viscera with the intestine content (except for liver and pancreas) was washed with tap water and shrouded in to smaller pieces using a sterilized blade as demonstrated by Taskin and Kurbanoglu [7] and Lasekan et al [9]. Next the viscera was grinded intermittently for 1 h using a blender (Panasonic MX- GM1011) and kept at −20 °C for future used. The raw grinded viscera was analyzed for elemental composition, dry matter, crude protein, crude fat, sugars and ash contents.

### Hydrolysis of the processed chicken viscera

**Fig. 1:**
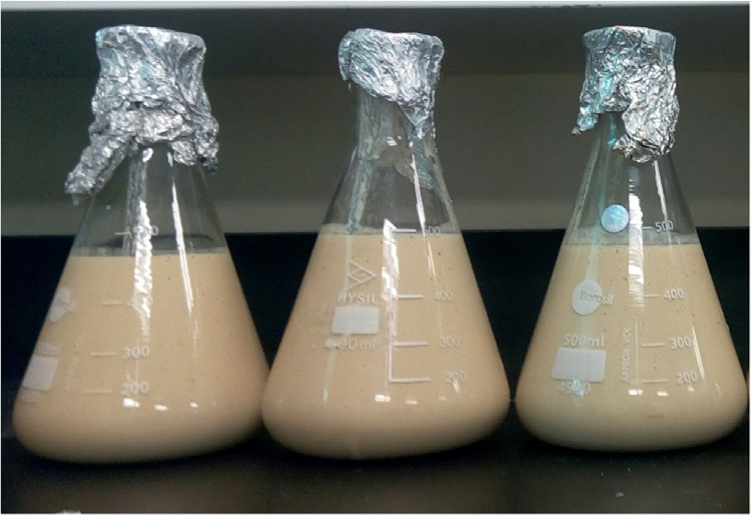
Chicken viscera homogenate.

The chicken viscera was hydrolyzed using acid hydrolysis in accordance with modified methods demonstrated by Jamdar and Harikumar [2] and Zhu et al [8]. Briefly, 40% homogenate of the grinded viscera (Fig. 1) was prepared using sterile water. The initial pH of the homogenate (pH 6.5) was adjusted to 2.8 using 1 N HCl. About 60 mL of 1 N HCl was needed to shift down the pH of 1 L of homogenate (40%) to 2.8. The homogenate was subsequently allowed to hydrolyze for 6 h at 55 °C with constant stirring at 150 rpm. Upon completion of 6 h, the hydrolyzed samples were dispensed into 250 mL tubes and centrifuged at 10,000 rpm using giant Centrifuge (KUBOTA 5922). The supernatant was carefully collected into sterile Scott bottle as the hydrolysate. The pH of collected supernatant was adjusted with 4 M NaOH. The sample was finally autoclaved for 20 min at 121 °C. The hydrolysate was freeze dried and analyzed for elemental composition, crude protein and heavy metals.

### Optimization of culture conditions

The culture conditions for growth of any microorganism has significant effect on its bioflocculant production or flocculation rate of the bioflocculant [3]. In an attempt to decide suitable culture conditions for growing *A. flavus* and bioflocculant yield with the viscera hydrolysate, five conditions were considered at a time. These conditions include; time (0– 72 h), agitation speed (50–200 rpm), pH (4–9), temperature (25–40 °C) and inoculum size (2%–10%). In order to study a particular condition, others were fixed while the condition of interests was varied accordingly. In subsequent experiments, the best values found from the studied conditions were used as the constant values until each of all the parameters were studied at a time.

### Bioflocculant purification and flocculation efficiency

The purification of the bioflocculant was carried out following the techniques demonstrated by Salehizadeh, Vossoughi [4] and Xiong, Wang [5]. The rich culture supernatant was mixed with cold ethanol (95%), at ratio of 1:2 culture supernatant-ethanol and kept at 4 °C for 8 h. The mixture was further centrifuged at 10,000 rpm, 30 min, 4 °C and the resulting residue liquefied in deionized water at ratio 1:2 (v/v). This process was repeated twice before the purified bioflocculant was lyophilized and vacuum dried.

**Fig. 2:**
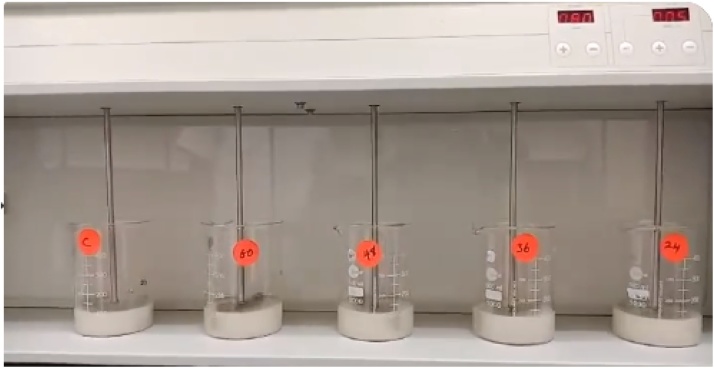
Bioflocculation test.

The flocculation efficiency of the bioflocculant was estimated according to the techniques of Kurane et al. and Xiong et al. Briefly, 2 mL of crude bioflocculant and 3 mL of 1% CaCl_2_ were mixed with 100 mL of Kaolin clay solution (4 g/L) in 500 mL beaker and the pH adjusted to 7.0 ± 0.1. The beaker containing the mixture was mounted to a flocculator (JLT 6 Velp Scientifica, Italy) (Fig. 2) and stirred at 200 rpm for 1 min followed by 80 rpm for 5 min and finally held still for 5 min. The absorbance of the upper part of the mixture and the control (where 2 mL sterile medium was used in place of the bioflocculant) were measured at 550 nm with T60 spectrophotometer. The flocculation efficiency was calculated and expressed in percentage using the formula in Eq. (1) [6].

(1) Flocculation efficiency = [(*A* – *B/A*) × 100%]

Where A represent the absorbance of the control at 550 nm and B represents absorbance of the sample at 550 nm.

### Effectiveness of the hydrolysate for growth of *A. flavus* and bioflocculant yield

The hydrolysate was characterized to contain the components listed in Table 1. The various culture conditions mentioned above were tested for the growth of *A. flavus* and their bioflocculant yield. Prior to tests, a bioflocculant producing strain; *A. flavus* S44-1 was reactivated from glycerol stock. To prepare the inoculums, *A. flavus* was grown on PDA and incubated at 30 °C for 5 days. The culture plate containing *A. flavus* spores was flooded with appropriate 1% Tween 80. The spores were then scraped into a sterilized flask with the aid of a sterile glass hockey stick. The spores were counted with the aid of haemocytometer and adjusted to 1.0 × 10^6^ spore/mL. Appropriate volume of the spore was inoculated into 50 mL liquid viscera hydrolysate in 250 mL flask and incubated at the temperature of interest, time and shaker speed depending on the culture condition under study.

**Table 1:** Composition of the Viscera Hydrolysate.

Parameters	content
Crude Protein, %w/w	5.40
Sugar, %w/w	3.20
Carbon (C), %w/w	5.86
Nitrogen, %w/w	1.27
Carbon : Nitrogen	5.08
Sulphur (S), %w/w	0.83
Hydrogen (H) %w/w	10.07
Heavy metals	
Lead (Pb) ppm	Not detected
Arsenic (As) ppm	Not detected
Mercury (Hg), ppm	Not detected
Cadmium (Cd), ppm	Not detected

**Fig. 3:**
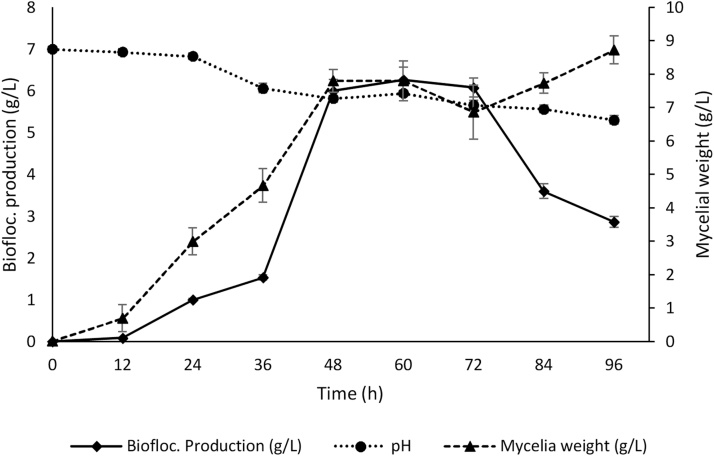
Time course of bioflocculant production and Mycelial weight of *A. flavus* using chicken viscera hydrolysate as a sole medium.

**Fig. 4:**
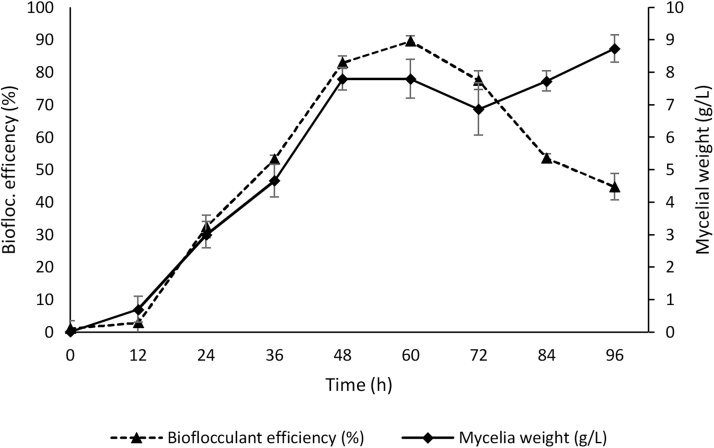
Time course of bioflocculant flocculation efficiency and Mycelial weight from *A. flavus* using chicken viscera hydrolysate as a sole medium.

The best culture conditions for *A. flavus* growth and bioflocculant yield were incubation time of 72 h, pH 7, shaker speed 150 rpm, temperature 35 °C and inoculum 4%. The bioflocculant yield and its efficiency were both parallel to mycelia weight at the logarithm and stationery phases of *A. flavus* growth profile (Fig. 3, Fig. 4). However, there was decline in production and efficiency beyond 72 h even with continuous increase mycelia weight. This is partly due to synthesis of bioflocculant degrading enzymes.

Although bioflocculants have generally been reported to be safe [1], the ability of fungi to produce toxin coupled with paucity of information on evaluation of safety of bioflocculant produced by microorganisms make it important to evaluate the toxicity of the bioflocculant of this nature prior to scale up. Also, there are many bioflocculant purification methods, most of which are relatively cheap, exploration of cost effective purification method that can yield highly efficient flocculant is in addition to used of low cost substrate a key factor to be considered in scaling up the process of bioflocculant production. Once these conditions are met the scale up process become more viable.
