# Supplementary material for: Development of a new culture medium for bioflocculant production using chicken viscera
Source: MethodsX. 2019 Jun 13;6:1467–72. doi: 10.1016/j.mex.2019.06.002 (PMC6593163; doi:10.1016/j.mex.2019.06.002)
Supplement: Supplementary file 1 [file mmc1.docx]

**Supplementary material and/or Additional information:**

**Background information**

The major required component of a microbial medium includes carbon, nitrogen, sulphur and phosphorus. These requirements constitute the main part of the production-cost for the microbial bioproduct synthesis. Among the substrates, nitrogen source is the most exorbitant component [[7](#_ENREF_7)]. Commercially, the nitrogen sources are synthesized from valuable and relatively expensive sources thereby adding to the cost of media for microbial bioproduct synthesis. Due to the high costs of commercial nitrogen sources, the cheap renewable sources from plant and animal waste are investigated. In Malaysia as well many developed and developing countries, poultry industry holds an essential position in the global sources of meat production. At the same time, a high amount of carbon and nitrogen sources such as feathers, viscera, heads, legs and bones are accumulated in form of waste [[8](#_ENREF_8)]. These poultry waste contains both carbon and nitrogen sources needed for microbial growth and bioproducts synthesis. The viscera account for up to 30% of the total waste while feather constitute close to 10%. Though they are sometimes used for processing of feedstuff for livestock, fertilizers and pet foods, a significant amount is being discarded. However, improper disposal could cause environmental challenges in form of pollution, diseases and loss of waste of important bioresources such as proteins, enzymes and lipids [[9](#_ENREF_9)]. Therefore, alternative ways of managing these wastes will no doubt reduce environmental and health risks to residents.

Further, the chemical flocculants used for waste water treatment and industrial process are associated with secondary pollutant accumulation. As a result, there is ongoing research and industrial effort to exploit production of flocculant from microorganisms however the cost of production of bioflocculants constitute a major setback. In line with the search for low-cost substrate for bioflocculant production, we focused on developing a suitable media for bioflocculant production. This work contributes to the ongoing interest of producing bioflocculant from waste as substitute to exorbitant commercial media that increases the cost of bioflocculant production. It brings into view the invention of new culture media for bioflocculant production and a viewpoint for possibility of this innovation for imminent prospect of large scale bioflocculant production from chicken viscera and possibly for commercialization. The success in establishing substitution of commercial nutrient sources with chicken viscera for bioflocculant production offer a promising scientific wastewater treatment alternative that is cost-effective and eco-friendly while also combating environmental pollution from chicken viscera.

**Failed method**

Chicken viscera was collected from wet market (Ayam Kempas Sdn Bhd, Johor). The viscera were immediately transported to the laboratory in iced condition. The parts of the viscera such as liver, spleen and pancreases not needed for this study were carefully removed. The chicken viscera with the intestine content was washed with tap water and shrouded in to smaller pieces using a sterilized blade. Next the viscera was grinded intermittently for 1 h using a blender (Panasonic MX- GM1011) and kept at -20°C for future used. The raw grinded viscera were analyzed for elemental composition, Dry Matter, Crude Protein, Crude Fat, sugars and ash contents. The same procedure was followed for chicken viscera which had the intestine content drained off.

To utilize the viscera for bioflocculant production, different amount of the blended viscera was directly dissolved in a conical flask containing 100 mL sterile deionized water. The pH was adjusted as required before inoculation with *A. flavus*. The flask was incubated at the temperature and shaking speed of interest. The culture broth was further filtered using vacuum pump. The filtrate was used as the crude bioflocculant for flocculation of Kaolin suspension and for bioflocculant purification following the procedures outlined in the method above. The residue collected on the filter paper was used for biomass estimation. This method yielded very little or no flocculation and bioflocculant. There was also challenges of mycelia weight estimation as the filtrate was visibly found to contain undissolved viscera particles. There were also issues with the sterilization of the blended viscera as it appears to be cooked when autoclaved whereas UV sterilization was not helpful. The presence of undissolved viscera particles as part of biomass and no bioflocculant recovery from the culture supernatant prompted our insinuation that the nutrient in the viscera were not available for fungi used. On that account we come up with the new method outlined in this article
